# A simulation framework for reciprocal recurrent selection-based hybrid breeding under transparent and opaque simulators

**DOI:** 10.3389/fpls.2023.1174168

**Published:** 2023-06-27

**Authors:** Zerui Zhang, Lizhi Wang

**Affiliations:** ^1^ Program of Bioinformatics and Computational Biology, Iowa State University, Ames, IA, United States; ^2^ Department of Industrial and Manufacturing Systems Engineering, Iowa State University, Ames, IA, United States; ^3^ Department of Statistics, Iowa State University, Ames, IA, United States

**Keywords:** hybrid breeding, reciprocal recurrent selection, opaque simulator, genomic prediction, genomic selection

## Abstract

Hybrid breeding is an established and effective process to improve offspring performance, while it is resource-intensive and time-consuming for the recurrent process in reality. To enable breeders and researchers to evaluate the effectiveness of competing decision-making strategies, we present a modular simulation framework for reciprocal recurrent selection-based hybrid breeding. Consisting of multiple modules such as heterotic separation, genomic prediction, and genomic selection, this simulation framework allows breeders to efficiently simulate the hybrid breeding process with multiple options of simulators and decision-making strategies. We also integrate the recently proposed concepts of transparent and opaque simulators into the framework in order to reflect the breeding process more realistically. Simulation results show the performance comparison among different breeding strategies under the two simulators.

## 1 Introduction

Hybrid breeding typically refers to breeding among genetically diverse pure line populations to harvest hybrid progeny F1 that have superior performance in certain favorable traits over their inbred parents. This phenomenon is known as heterosis. The concept was validated by some early recorded experiments (Shull, 1908; Shull, 1909). A number of economically important species have benefited from hybrid breeding, including maize, rice, and sorghum (Fu et al., 2014; Labroo et al., 2021). However, the mechanism has not yet reached a consensus and there are three possible hypotheses for the explanation of overperformance of hybrid offspring. The dominance hypothesis states that heterosis is due to dominant alleles from either parent cancelling the effect of deleterious recessive alleles contributed by the other parent in the hybrid (Davenport, 1908; Bruce, 1910; Jones, 1917). The overdominance hypothesis attributes heterosis to the fact that heterozygous genotypes are more adaptive than homozygous ones on a single locus (Shull, 1908; Shull, 1909). The epistasis hypothesis attributes the contribution of positive epistatic interactions between non-allelic genes to heterosis (Minvielle, 1987).

Hybrid breeding attempts to take advantage of dominance effects (Hallauer et al., 2010) by breeding for inbred parents whose F1 progeny will possess positive heterosis. To address the assessment and selection for heterosis, hybrid breeding usually makes use of self-pollinated or double-haploid inbred lines, followed by progeny evaluation in heterotic pools (Fritsche-Neto et al., 2021). As such, hybrid breeding involves both inter-population breeding and intra-population breeding (Labroo et al., 2021). There are three main steps in hybrid breeding: (1) selecting founders of heterotic pools, (2) crossing parental lines within heterotic pools, and (3) selecting breeding parents for offspring production (Labroo et al., 2021). As a representative approach, reciprocal recurrent selection (RRS) was pioneered to help develop selective maize recombinant lines featuring the heterosis selection (Robinson et al., 1949). It is a cyclical breeding procedure designed to improve the cross of two populations from different heterotic groups, where genotypes from two homozygous populations are evaluated in reciprocal crosses and the best-adapted genotypes of each population are selected and recombined to give rise to improved hybrid (Santos et al., 2005; Li et al., 2008).

Owing to the resource-intensive and time-consuming nature of the RRS process, it is challenging to design, validate, and compare algorithms for the many decisions to be made in RRS. As a result, it becomes important to use a simulation framework that can quickly and realistically simulate the process, as alluded to in Labroo et al. (2022) and Powell et al. (2020). An ideal framework should consist of simulation modules (e.g., phenotyping, genotyping, and meiosis) and decision-making modules (e.g., genomic prediction and genomic selection); to address heterosis in hybrid selection, dominance effects should also be considered in the decision-making modules. Existing simulation tools for plant breeding that build upon diverse mechanisms include AlphaSimR (Gaynor et al., 2021), AlphaSim (Faux et al., 2016), QM Sim (Sargolzaei and Schenkel, 2009), MoBPS (Pook et al., 2020), XSimV2 (Chen et al., 2022), and MBP (Gordillo and Geiger, 2008). Breeders can obtain genotype and/or phenotype at the individual or population level after providing inputs such as the numbers of chromosomes and loci and quantitative trait loci (QTL), minor allele frequency (MAF) of each locus, mutation rates, heritability, and pedigree (Pook et al., 2020). The implemented selection methods are mainly truncation selection based on different criteria such as phenotypes, genetic values, breeding values, or estimated breeding values, without directly accounting for dominance effects.

In this paper, we performed comparisons among different breeding strategies under the simulation framework for RRS using transparent and opaque simulators. The concepts of transparent and opaque simulators were formally defined and formulated in Amini et al. (2021) for genomic selection. In Gaynor et al. (2021), similar concepts were implemented in the AlphaSimR with multiple genetic effects and genomic prediction and selection methods. The defining feature of a transparent simulator is the simplifying assumption that the observed genomic data, which are used by the decision-making modules for genomic prediction and genomic selection, are the complete genomic information that, together with the environment, contributed to the determination of phenotype. In contrast, an opaque simulator acknowledges the fact that the observed genomic data are only a subset of the whole genomic information, and the unobserved genomic information also contributes to the determination of phenotype. Since opaque simulators intuitively reflect nature more accurately than transparent ones, we are curious to compare the performances of different genomic prediction and genomic selection algorithms under these two simulators.

## 2 Method

**Figure 1:**
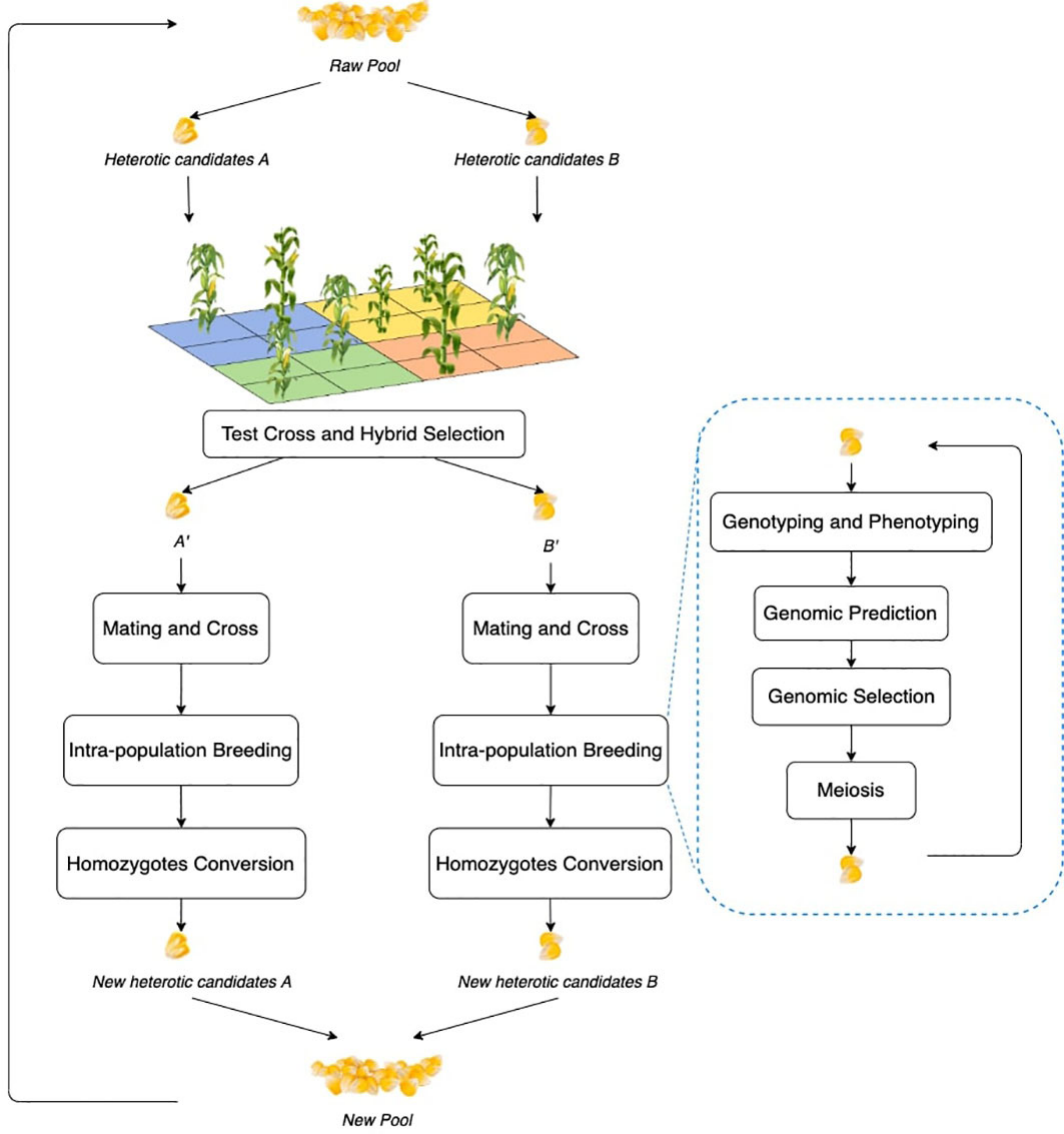
Workflow of RRS. Each block is a process step and the illustrations can be found in the following subsections. The blue bubble contains the process of general intra-population breeding, which is repeated within the overall RRS. Each step will be also expanded.

The workflow of the RRS simulation framework is illustrated in **Figure 1**, which has intra-population breeding as a sub-component (Hallauer et al., 2010; Labroo et al., 2021). Intra-population breeding refers to the common strategy in plant breeding to perform recurrent individual evaluation and crosses within a given pool of candidates.

As shown in **Figure 1**, RRS consists of five steps: (1) first select and divide the raw pool of individuals into two different groups, which become the heterotic candidates A and ℬ ; (2) mutually test cross A and ℬ , and perform hybrid selection to identify $A^{'}$ and ${ℬ}^{'}$ that contribute to heterosis; (3) mate and cross the elites to enhance genetic diversity; (4) let $A^{'}$ and ${ℬ}^{'}$ go through intra-population breeding to exploit genetic gains; and (5) convert the heterozygotes to homozygotes by doubled haploid or self-crossing and prepare for the next cycle. Major modules of the RRS workflow include test cross and hybrid selection, mating and cross, intra-population breeding (including genotyping and phenotyping, genomic prediction, genomic selection, and meiosis), and homozygote conversion, which will be explained in detail in the following subsections.

### 2.1 Nomenclature

Here, we define the notations used in this paper.

**Table:** 

N	Number of the individuals in a population, a scalar
L	Number of SNPs of an individual, a scalar
G	Genotype of a population, a binary matrix $G\in B^{L\times N\times 2}$ , with element $G_{i,j,m}$ indicating whether the allele in the 1st ( m=1 ) or 2nd ( m=2 ) chromosome of diploid individual i at locus j is a major allele ( $G_{i,j,m}=1$ ) or a minor allele ( $G_{i,j,m}=0$ )
α	Additive effect, a vector $\alpha \in {\mathbb{R}}^{L}$ , with $\alpha_{j}$ being the allele effect for locus j
β	Dominance effect, a vector $\beta \in {\mathbb{R}}^{L}$ , with $\beta_{j}$ being the allele effect for locus j
r	Recombination frequencies, a vector $r\in {\mathbb{R}}^{L-1}$ , with $r_{j}$ being the recombination frequency between loci j and j+1
v	Genetic estimated breeding values (GEBVs), a vector $v\in {\mathbb{R}}^{N}$ , with $v_{i}$ being the GEBV of individual i

We further define the indicator of accumulating additive effects at the j th locus of the i th individual as


(1) $$g_{i,j}=G_{i,j,1}+G_{i,j,2}i=1,\cdots ,N;\;j=1,\cdots ,L.$$

and use $d_{i,j}$ to indicate heterozygosity at the j th locus of the i th individual as


(2) $$d_{i,j}=I\left(g_{i,j}=1\right)i=1,\cdots ,N;\;j=1,\cdots ,L.$$

Define the hidden genotypic information as $g_{i,q}^{'}$ and $d_{i,q}^{'}$ for q={1,2,⋯,Q} and the corresponding additive effects $\alpha_{q}^{'}$ and dominance effects $\beta_{q}^{'}$ . Let $\bar{G}$ denote the whole genome, which is a mixture of partially observed genotypes G and the hidden information $G^{'}$ . Let $\bar{r}$ denote the recombination frequency for $\bar{G}$ . The evaluation metric mainly used in the article is GEBV and we can calculate it for the i th individual as


(3) $$v_{i}=\sum_{j=1}^{L}\alpha_{j}g_{i,j}+\sum_{j=1}^{L}\beta_{j}d_{i,j}$$

For the phenotypic values $p=\left(p_{i}\right)$ , we further define $e=\left(e_{i}\right)$ as the corresponding environmental effects where $e_{i}∼N\left(0,\sigma_{e}^{2}\right)$ . Assume the phenotypic values are composed of genotypic and environmental effects so that we have


(4) $$p_{i}=\sum_{j=1}^{L}\alpha_{j}g_{i,j}+\sum_{j=1}^{L}\beta_{j}d_{i,j}+e_{i}$$

The variance for the environmental effects is controlled by broad-sense heritability $H^{2}$ and may be subject to changes in different breeding cycles and can be shown in the following equation:


(5) $$H^{2}:=\frac{Var\;(Genotype)}{Var\;(Phenotype)}=\frac{Var\;(p-e)}{Var\;(p)}$$

(6) $$\sigma_{e}^{2}=\frac{\sum_{i=1}^{n}{\left(p_{i}-\bar{p}\right)}^{2}}{n-1}\left(1-H^{2}\right).$$

### 2.2 Genotyping and phenotyping

Genotyping is the process of obtaining genomic information, and phenotyping is the evaluation of traits of interest of plant individuals. We describe the simulation of genotyping and phenotyping steps using transparent and opaque simulators as follows.

* A conventionally used **transparent simulator** makes two major assumptions: (1) the whole genome of an individual contains no more information than what is revealed by the genetic markers and (2) the true genetic effects ( $\alpha_{j}$ and *β_j_*) for all markers are the same as the results from the genomic prediction. As such, the phenotypic value of individual i is determined as


(7) $$p_{i}=v_{i}+e_{i}=\sum_{j=1}^{L}\alpha_{j}g_{i,j}+\sum_{j=1}^{L}\beta_{j}d_{i,j}+e_{i}$$

where $\alpha_{j}$ and $\beta_{j}$ are, respectively, additive and dominance genetic effects of allele j , and $e_{i}$ is a random error term for individual i .

* In the proposed **opaque simulator**, both assumptions made in transparent simulators are relaxed. A separate genotype is assumed to represent the ground truth genome, which is a superset of the observed genotype; the phenotypic value is determined by the whole genome, whose ground truth genetic effects are never revealed to the genomic prediction module. As such, the phenotypic value of individual i is determined as


(8) $$p_{i}=v_{i}+e_{i}=\sum_{j=1}^{L}\alpha_{j}g_{i,j}+\sum_{j=1}^{L}\beta_{j}d_{i,j}+\sum_{q=1}^{Q}\alpha_{q}^{'}g_{i,q}^{'}+\sum_{q=1}^{Q}\beta_{q}^{'}d_{i,q}^{'}+e_{i},$$

where $\alpha_{q}^{'}$ and $\beta_{q}^{'}$ are, respectively, additive and dominance genetic effects of hidden allele q that exists in the ground truth but unobservable by other modules.

### 2.3 Genomic prediction

Genomic prediction is a technology that builds the quantitative relationships between phenotypic responses p and the SNP information G , and predicts GEBV $\hat{v}$ to guide the computation-assisted selection. The sequenced and phenotyped group are often treated as the sample for effect estimation. Three predictors are considered in this paper.

* **Bayesian predictor**. We use the following Bayesian linear mixed model (P´erez and de los Campos, 2014; Lopes et al., 2015) to carry out the estimation based on the observable genotypes G :


(9) $$p_{i}=\mu +\sum_{j=1}^{L}a_{j}g_{i,j}+\sum_{j=1}^{L}b_{j}d_{i,j}+\epsilon_{i},$$

$$a_{j}\left|\sigma_{a}^{2}∼N\left(0,\sigma_{a}^{2}\right),b_{j}\right|\sigma_{d}^{2}∼N\left(0,\sigma_{d}^{2}\right),\epsilon_{i}∼N\left(0,\sigma_{\epsilon }^{2}\right)$$

where μ is the mean value within the group and $\epsilon_{i}$ is the random error for individual i with mean zero and a fixed variance $\sigma_{\epsilon }$ . Each additive effect $a_{j}$ and dominance effect $b_{j}$ are assumed a normal distribution with mean zero and a fixed variance denoted by $\sigma_{a}^{2}$ and $\sigma_{b}^{2}$ , respectively. With estimated $\hat{\mu }$ , $\hat{a_{j}}$ , and $\hat{b_{j}}$ , the estimated GEBV $\hat{v_{i}}$ for individual i is then calculated as


(10) $$\hat{v_{i}}=\hat{\mu }+\sum_{j=1}^{L}\hat{a_{j}}g_{i,j}+\sum_{j=1}^{L}\hat{b_{j}}d_{i,j}.$$

* **Perfect predictor**. This represents an ideal prediction algorithm that is able to perfectly estimate the ground truth GEBV for any individual i :


(11) $$\hat{v_{i}}=v_{i}.$$

Depending on whether a transparent or opaque simulator is used, $v_{i}$ takes the definition in either Equation (7) or (8), respectively.

* **Phenotypic predictor**. This predictor simply uses the observed phenotype as the estimated GEBV:


(12) $$\hat{v_{i}}=p_{i}.$$

### 2.4 Genomic selection

The goal of the genomic selection module is to select breeding parents based on genotypic and/or phenotypic information. Here, we consider the widely used truncation selection, which selects individuals with the highest GEBVs as breeding parents. This selection algorithm can be formulated as the following optimization model:


(13) $$\text{maximize }\sum_{i=1}^{N}x_{i}{\hat{v}}_{i}$$

(14) $$\text{subject to }\sum_{i=1}^{N}x_{i}=M$$

(15) $$x_{i}\in \left\{0,1\right\},i=1,\ldots ,N.$$

where binary decision variable $x_{i}$ indicates whether individual i is selected ( $x_{i}=1$ ) or not ( $x_{i}=0$ ), and M is the number of individuals to be selected.

### 2.5 Meiosis

When two individuals $i_{1}$ and $i_{2}$ are crossed, the genotype of their progeny is simulated using the cross(·) function, which has the same procedures as the reproduce step in Goiffon et al. (2017). The output of cross(ot)cross(ot)cross(·) can be viewed as an offspring conceived from one chromosome provided by each parent and the recombinations controlled by r .

Let matrix $G=\left\{G_{i,j,m}\right\}$ for i={1,⋯,N}, j={1,⋯,L}, m={1,2} denote SNP of all haplotype blocks for the transparent simulator and $\bar{G}=\left\{{\bar{G}}_{i,j^{'},m}\right\},\;j^{'}=\left\{1,\cdots ,\bar{L}\right\}$ for the opaque simulator. Vectors of recombination rates $r=\left\{r_{j}\right\}$ for j={1,⋯,L} and $\bar{r}=\left\{{\bar{r}}_{j^{'}}\right\}$ for $j^{'}=\left\{1,\cdots ,\bar{L}\right\}$ match the length of SNPs for G and $\bar{G}$ . Note that G was employed in the transparent simulator and was also taken as the set of markers in the opaque simulator; $G^{'}$ was the hidden genomic information and was only used in the opaque simulator. The relationship can be established as $\bar{G}=\left\{G,G^{'}\right\}$ . The application of cross(·) for the transparent and opaque simulator can be viewed as


(16) $$G^{t+1}=\text{cross}\left(G_{i_{1}}^{t},G_{i_{2}}^{t},r\right),$$

(17) $${\bar{G}}^{t+1}=\text{cross}\left({\bar{G}}_{i_{1}}^{t},{\bar{G}}_{i_{2}}^{t},\bar{r}\right).$$

### 2.6 Test cross and hybrid selection

Test cross is a nontrivial step to provide the heterogeneous progeny for further hybrid breeding. Let us interpret the process with the following matrix notation: assume the test cross has been executed mutually between N homozygous individuals from population A and ℬ , denoted as $\{a_{1},a_{2},\cdots ,a_{N}$ } and $\left\{b_{1},b_{2},\cdots ,b_{N}\right\}$ , respectively. The indices for hybrids are therefore denoted as $\left(a_{k},b_{l}\right)$ , where k={1,⋯,N} and l={1,⋯,N} . We use $v_{a_{k},b_{l}}$ to denote the GEBV of hybrid with $a_{k}$ and $b_{l}$ as parents so that the GEBV matrix V for the test cross hybrids can be written with rows of $a_{k}$ and columns of $b_{l}$ as


$$\begin{array}{cc} & \begin{array}{c} \begin{array}{cccc} \begin{array}{cc} b_{1} & b_{2} \end{array} & \cdots & b_{N-1} & b_{N} \end{array} \end{array}\\ V\;=\begin{array}{c} a_{1}\\ a_{2}\\ \vdots \\ \begin{array}{c} a_{N-1}\\ a_{N} \end{array} \end{array} & \left(\begin{array}{c} \begin{array}{c} \begin{array}{cccc} \begin{array}{cc} v_{a_{1},b_{1}} & v_{a_{1},b_{2}} \end{array} & \cdots & v_{a_{1},b_{N-1}} & v_{a_{1},b_{N}} \end{array}\\ \begin{array}{cccc} \begin{array}{cc} v_{a_{2},b_{1}} & v_{a_{2},b_{2}} \end{array} & \cdots & v_{a_{2},b_{N-1}} & v_{a_{2},b_{N}} \end{array} \end{array}\\ \begin{array}{cccc} \begin{array}{cc} \vdots & \vdots \end{array} & \vdots & \vdots & \vdots \end{array}\\ \begin{array}{cccc} \begin{array}{cc} v_{a_{N-1},b_{1}} & v_{a_{N-1},b_{2}} \end{array} & \cdots & v_{a_{N-1},b_{N-1}} & v_{a_{N-1},b_{N-1}} \end{array}\\ \begin{array}{cccc} \begin{array}{cc} v_{a_{N},1} & v_{a_{N},2} \end{array} & \cdots & v_{a_{N},b_{N-1}} & v_{a_{N},b_{N}} \end{array} \end{array}\right) \end{array}$$

Note that the above GEBV matrix V is actually realized by a specified predictor so that all the values within cell of $\hat{V}$ would be ${\hat{v}}_{a_{k},b_{l}}$ .

Given the results of test cross, genomic selection aims to identify individuals from one population that have exhibited promising combining ability with those from the other population. Therefore, the compromise of dominance effects in addition to additive effects is the concern for genomic selection. Based on the two populations A and ℬ and their hybrid GEBV matrix $\hat{V}$ , our goal is to select 2K (2K<N) individuals from each to get two groups $A^{'}$ and ${ℬ}^{'}$ featuring good hybrids with high GEBVs.

We describe here two common strategies for practical applications and show the difference in their focus on hybrid breeding in optimization, i.e., the different ways of evaluating the $\hat{V}$ matrix. Note that the following formulations are based on genomic selection on A so that we focus on the rows of matrix $\hat{V}$ . The formulations can be adjusted to apply to the genomic selection on ℬ when switching to the columns of $\hat{V}$ .

* **General combining ability (GCA)**. GCA is designed to measure the average performances of test cross as evaluation over the row-wise (or column-wise) means of GEBV matrix $\hat{V}$ . It can reflect the general combining pattern between inbred lines from two populations.


(18) $$\text{maximize}\sum_{k=1}^{N}x_{k}\sum_{l=1}^{N}\frac{{\hat{v}}_{a_{k},b_{l}}}{N}$$

(19) $$\text{subject to }\sum_{k=1}^{N}x_{k}=2K,$$

(20) $$x_{k}\in \left\{0,1\right\},k=1,\ldots ,N.$$

* **Specific combining ability (SCA)**. SCA is designed as the evaluation of the row-wise (or column-wise) maximum of GEBV matrix $\hat{V}$ , which focuses more on the top performer contributed by dominance effects. To give the formulations, we define decision variables $y_{k,l}\in \left\{0,1\right\}$ in the form of a matrix Y with the same dimensions as the test cross GEBV matrix $\hat{V}$ , and it represents whether the crossing between $a_{k}$ and $b_{l}$ would be chosen (=1) or not (=0).


(21) $$\text{maximize }\sum_{k=1}^{N}\sum_{l=1}^{N}y_{k,l}{\hat{v}}_{a_{k},b_{l}}$$

(22) $$\text{subject to }\sum_{k=1}^{N}x_{k}=2K,$$

(23) $$\sum_{l=1}^{N}y_{k,l}=1,k=1,\ldots ,N,$$

(24) $$y_{k,l}\leq x_{k},k=1,\ldots ,N,$$

(25) $$y_{k,l}\leq x_{k},k=1,\ldots ,N,$$

(26) $$y_{k,l}\in \left\{0,1\right\},k=1,\ldots ,N,l=1,\ldots ,N.$$

### 2.7 Mating and cross

After the identification of homogeneous candidates that have the satisfying ability to reasonably compensate with individuals from the other genetically different population, the breeders need to cross the candidates by certain mating designs to improve their current GEBVs. We use $A^{'}$ as an example and the same strategy can be generalized to ${ℬ}^{'}$ . Assume the set $\tilde{A^{'}}=\left\{a_{\left(1\right)}^{'},a_{\left(2\right)}^{'},\cdots ,a_{\left(2K\right)}^{'}\right\}$ denotes the sorted individuals in decreasing order of GEBVs, then two designs are to be discussed.

* **Adjacent**. One direct way to produce progeny with high GEBV based on two superior parents, i.e., $a_{\left(1\right)}^{'}$ pairs with $a_{\left(2\right)}^{'}$ , $a_{\left(3\right)}^{'}$ pairs with $a_{\left(4\right)}^{'}$ , and so on until $a_{\left(2K-1\right)}^{'}$ pairs with $a_{\left(2K\right)}^{'}$ . Each pair is crossed to produce S heterozygous progeny.

* **Complementary**. Consider the possible complementary desirable alleles from parents so that mating with inferior ones may produce progeny with even higher GEBV, i.e., $a_{\left(1\right)}^{'}$ pairs with $a_{\left(K\right)}^{'}$ , $a_{\left(2\right)}^{'}$ pairs with $a_{\left(K+1\right)}^{'}$ , and so on until $a_{\left(K-1\right)}^{'}$ pairs with $a_{\left(2K\right)}^{'}$ . Each pair is crossed to produce S heterozygous progeny.

### 2.8 Conversion to homozygotes

The last step of one breeding cycle is the conversion from heterozygous individuals to homozygous so that another cycle of hybrid breeding could be initialized. Doubled haploid, which is the replication of one gamete, and self-cross can both achieve the goal.

## 3 Results

### 3.1 Simulation setting

This paper uses the same dataset as Moeinizade et al. (2019), which contains diploid SNP data for N=369 , $L_{0}=140,6757$ maize inbred lines. Recombination rates r are based on the genetic map developed from maize nested association mapping and is considered as “ground truth” for simulation and that errors of estimation have an equal effect on all selection methods (Yu et al., 2008).

To facilitate the simulation, we chose L=1,000 and $\bar{L}=10,000$ to extract markers and constructed haplotype blocks. The transparent simulator used those L=1,000 markers for both simulation and decision-making modules, whereas the opaque simulator used the same L=1,000 markers in the decision-making module but $\bar{L}=10,000$ SNPs in simulation modules. The comparisons are listed in **Table 1**. Vectors of recombination rates $r=\left\{r_{j}\right\}$ for j={1,⋯,L} and $\bar{r}=\left\{{\bar{r}}_{j^{'}}\right\}$ for $j^{'}=\left\{1,\cdots ,\bar{L}\right\}$ can be determined by either fixing the largest L−1 and $\bar{L}-1$ ones or using the water pipe algorithm (Han et al., 2017). To obtain homozygous individuals, for each individual within G and $\bar{G}$ , one gamete is randomly chosen and duplicated.

**Table 1:** Number of markers deployed in transparent and opaque simulators in the experiment setting.

	Transparent	Opaque
Simulation (phenotypes, meiosis)	1,000	10,000
Decision-making (prediction, selection)	1,000	1,000

**Figure 2:**
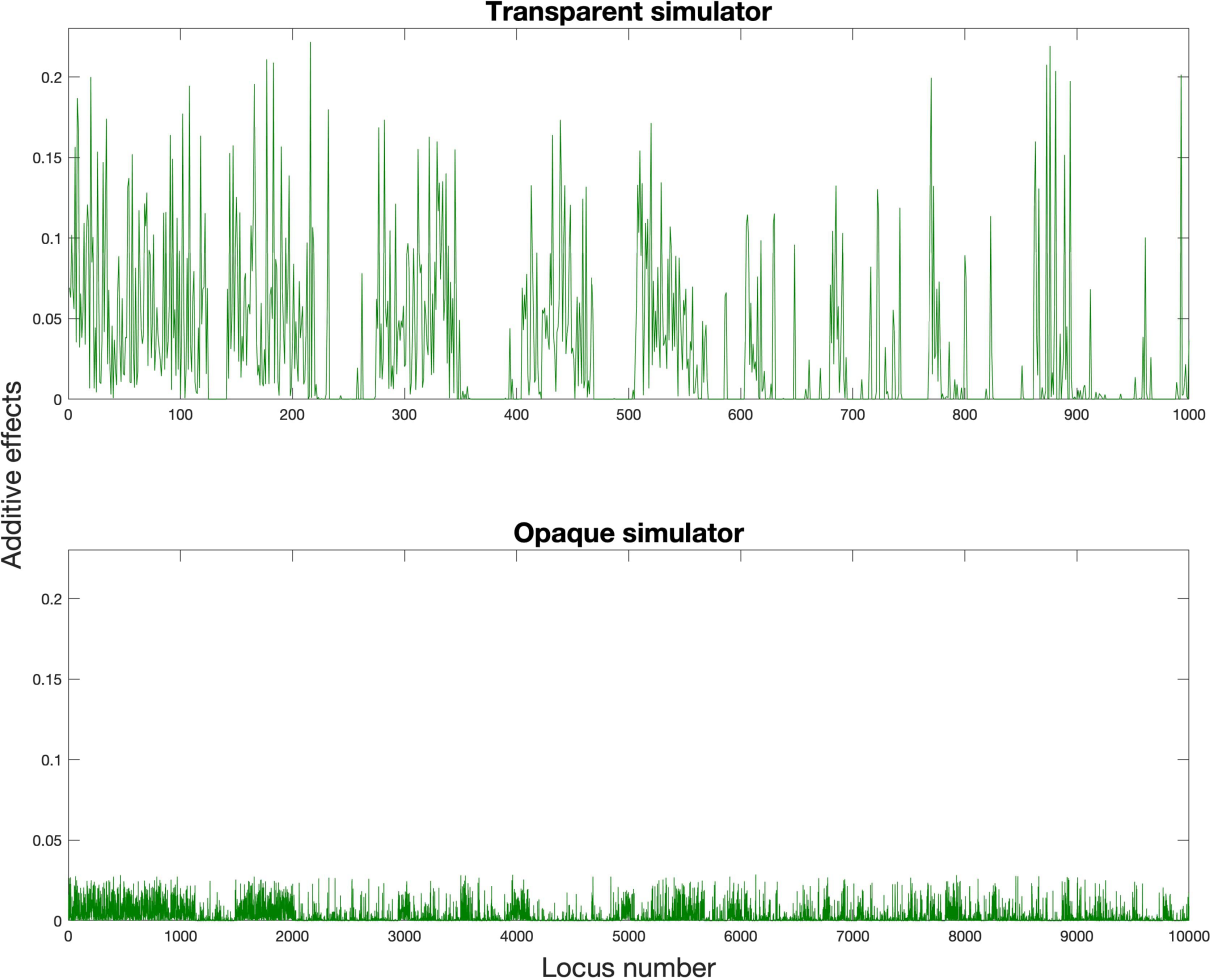
Assumed ground truth additive effects (green color, α and $\bar{\alpha }$ ).

**Figure 3:**
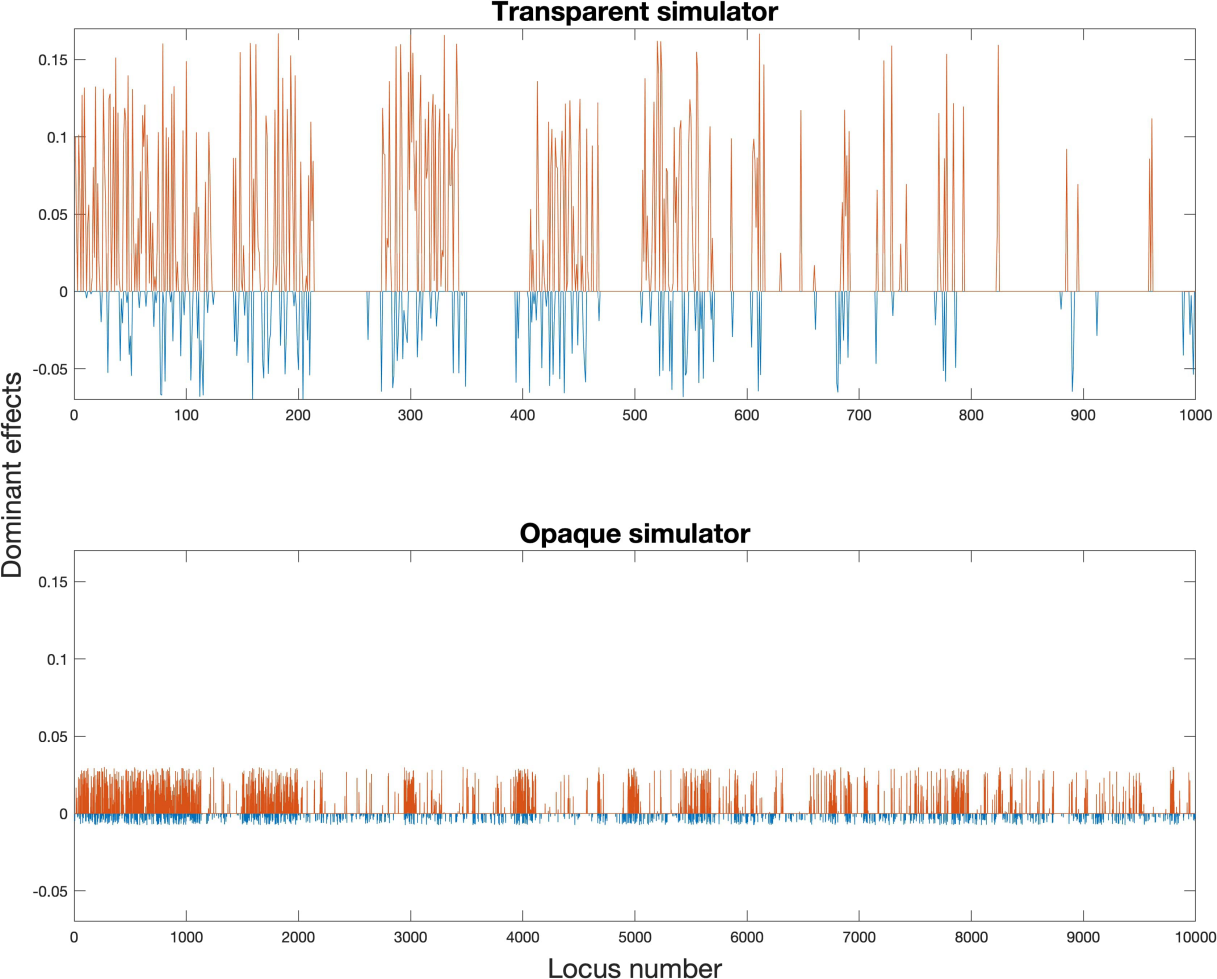
Assumed ground truth dominant effects (red for positive dominant effects, $\beta^{\left(+\right)}$ and ${\bar{\beta }}^{\left(+\right)}$ ; blue for negative dominant effects, $\beta^{\left(-\right)}$ and ${\bar{\beta }}^{\left(-\right)}$ ).

Vectors for the additive effects, $\alpha =\left\{\alpha_{j}\right\}$ and $\bar{\alpha }=\left\{{\bar{\alpha }}_{j^{'}}\right\}$ , positive dominance effects, $\beta^{\left(+\right)}=\left\{\beta_{j}^{\left(+\right)}\right\}$ and ${\bar{\beta }}^{\left(+\right)}=\left\{{\bar{\beta }}_{j^{'}}^{\left(+\right)}\right\}$ , and negative dominance effects, $\beta^{\left(-\right)}=\left\{\beta_{j}^{\left(-\right)}\right\}$ and ${\bar{\beta }}^{\left(-\right)}=\left\{{\bar{\beta }}_{j^{'}}^{\left(-\right)}\right\}$ , were set to satisfy the following equations:


$$\sum_{j=1}^{L}\alpha_{j}=\sum_{j^{'}=1}^{\bar{L}}{\bar{\alpha }}_{j^{'}}=30,$$

$$\sum_{j=1}^{L}\beta_{j}^{\left(+\right)}=\sum_{j^{'}=1}^{\bar{L}}{\bar{\beta }}_{j^{\prime }}^{\left(+\right)}=20,$$

$$\sum_{j=1}^{L}\beta_{j}^{\left(-\right)}=\sum_{j^{'}=1}^{\bar{L}}{\bar{\beta }}_{j^{'}}^{\left(-\right)}=-5.$$

and **Figures 2** and **3** showed our settings for the assumed ground truth genomic effects.

We designed 24 experiments and conducted simulations based on these settings to test the performance of framework. The layout for experiment settings is shown in **Table 2**, which traverses all distinct combinations of simulator, predictor, mating strategy, and genomic selection to compare the selection performances. Each experiment was repeated 100 times. Note that the parameter estimation of the Bayesian predictor was realized by using the BayesA model and the R package “BGLR” (P´erez and de los Campos, 291 2014). Each simulation consists of the following steps:

- **Step 1.** Randomly choose 200 individuals from the total of 369 and arbitrarily separate them into heterotic pool A and ℬ by the proposed heterotic separation algorithm.
- **Step 2.** Let A and ℬ go through RRS as shown in **Figure 1**.
- **Step 3.** Mutually cross the two new heterotic pools, and record and analyze the average GEBV of the top 100 hybrid offspring C as an evaluation of hybrid breeding.
- **Step 4.** Repeat **Step 2** and **Step 3** until the pre-specified cycle numbers are achieved. Here, we choose T=6 .

**Table 2:** Each experiment represented different combinations of simulator, predictor, mating strategy, and genomic selection for offspring performance comparison.

Experiment Index	Simulator	Predictor	Mating	Genomic Selection
1	Transparent	Perfect	Adjacent	GCA
2	Transparent	Perfect	Adjacent	SCA
3	Transparent	Perfect	Complementary	GCA
4	Transparent	Perfect	Complementary	SCA
5	Transparent	Phenotypic	Adjacent	GCA
6	Transparent	Phenotypic	Adjacent	SCA
7	Transparent	Phenotypic	Complementary	GCA
8	Transparent	Phenotypic	Complementary	SCA
9	Transparent	Bayesian	Adjacent	GCA
10	Transparent	Bayesian	Adjacent	SCA
11	Transparent	Bayesian	Complementary	GCA
12	Transparent	Bayesian	Complementary	SCA
13	Opaque	Perfect	Adjacent	GCA
14	Opaque	Perfect	Adjacent	SCA
15	Opaque	Perfect	Complementary	GCA
16	Opaque	Perfect	Complementary	SCA
17	Opaque	Phenotypic	Adjacent	GCA
18	Opaque	Phenotypic	Adjacent	SCA
19	Opaque	Phenotypic	Complementary	GCA
20	Opaque	Phenotypic	Complementary	SCA
21	Opaque	Bayesian	Adjacent	GCA
22	Opaque	Bayesian	Adjacent	SCA
23	Opaque	Bayesian	Complementary	GCA
24	Opaque	Bayesian	Complementary	SCA

We actually presented the “ground truth” GEBVs for all the results since all the authentic genomic effects are assumed and the true GEBV reflects the true enhancement of breeding on genomic effects.

### 3.2 GEBV comparisons for simulators, predictors, and mating

**Figure 4:**
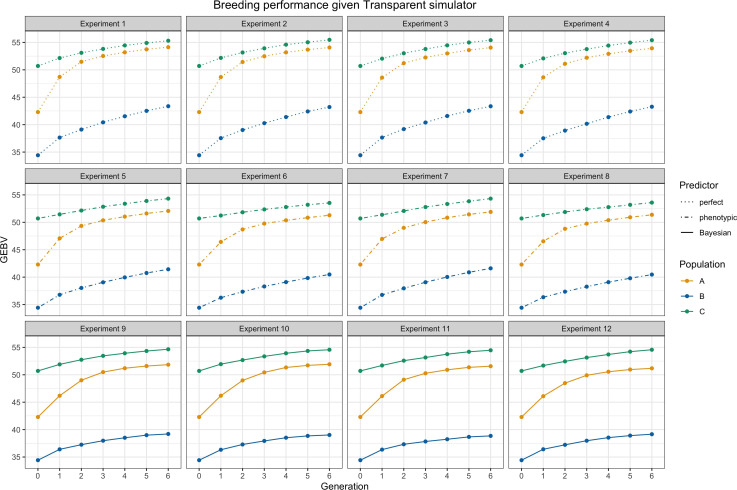
Breeding performance of experiments 1 to 12, i.e., the simulator is fixed as a transparent simulator. Each subtitle above the subfigure represents the experiment index. A and ℬ are parental populations and they are denoted by yellow and blue lines. C is the hybrid progeny denoted by green lines. The solid lines represent using Bayesian predictor, the dashed lines represent using perfect predictor, and the dot-dashed lines represent using phenotypic predictor. Parameter settings include $M=40,\;K=10,\;S=20,\text{ and }H^{2}=0.2$ .

**Figure 5:**
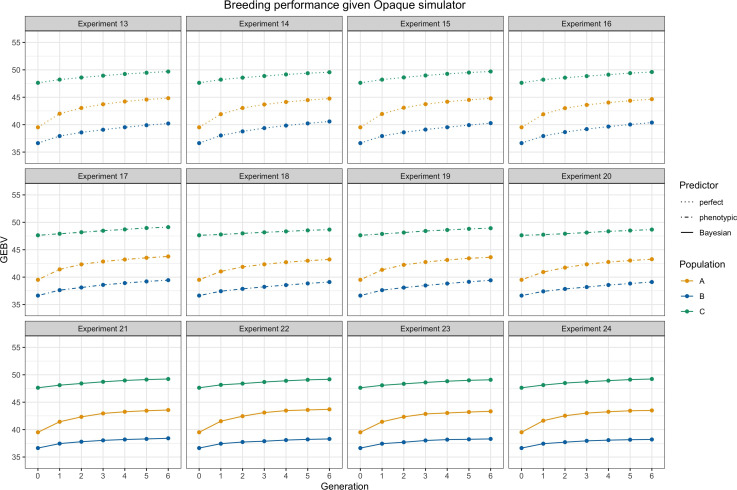
Breeding performance of experiments 13 to 24, i.e., the simulator is fixed as an opaque simulator. Each subtitle above the subfigure represents the experiment index. A and ℬ are parental populations and they are denoted by yellow and blue lines. C is the hybrid progeny denoted by green lines. The solid lines represent using Bayesian predictor, the dashed lines represent using perfect predictor, and the dot-dashed lines represent using phenotypic predictor. Parameter settings include $M=40,\;K=10,\;S=20,\text{and}\;H^{2}=0.2$ .

**Figure 6:**
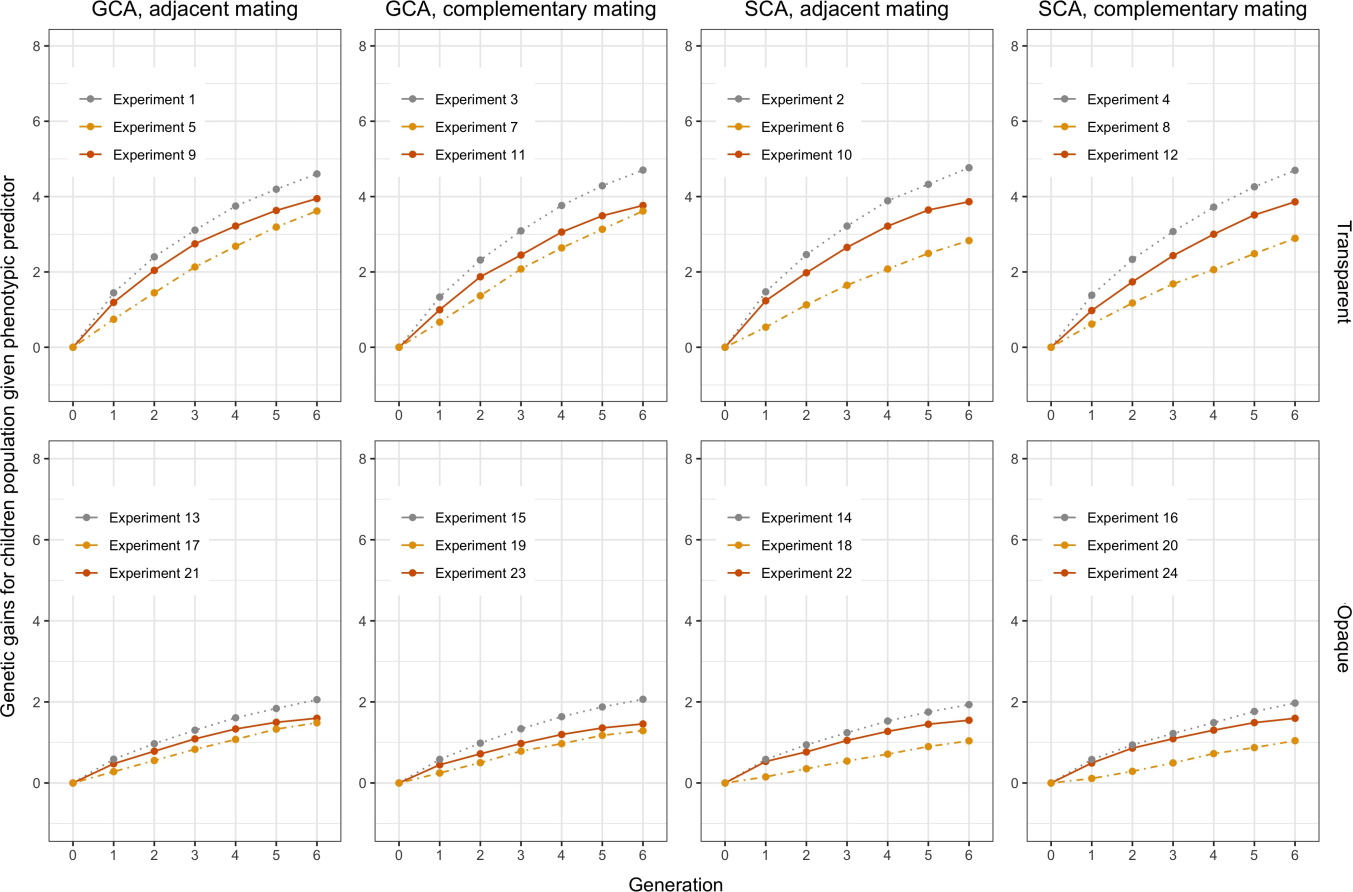
Genetic gain for the children group C computed from **Figures 4** and **5**. The orange solid lines represent using Bayesian predictor, the gray dashed lines represent using perfect predictor, and the yellow dot-dashed lines represent using phenotypic predictor.

We presented average GEBVs for two parental populations and their hybrid children population during T=6 breeding cycles under different settings through the simulations. **Figure 4** shows trends of average GEBVs of heterotic parental pools A and ℬ and the hybrid children C for each of the combinations consisting of three predictors, i.e., perfect, phenotypic, and Bayesian predicted; two mating strategies, i.e., adjacent and complementary; and two genomic selections, i.e., GCA and SCA, when the transparent simulator is fixed. **Figure 5** shows trends of GEBVs given the simulator is opaque. To zoom in the comparison among C specifically, **Figure 6** shows the genetic gains for children population C , which meant average GEBVs for each breeding cycle were subtracted from the baseline value in T=0 .

From **Figures 4** and **5** as a whole, we can conclude that the use of RRS was able to accomplish the goal of hybrid breeding: the trajectories of average GEBVs of A , ℬ , and C showed an increasing pattern in each experiment, and the GEBVs of hybrid progeny C were higher compared to their parents. In addition, we can see the overall influences of the transparent simulator and the opaque simulator on hybrid breeding; i.e., the GEBV growth of the three populations was much greater with the transparent simulator than with the opaque simulator. We believe that this is reasonable because the additive and dominance effects are orders of magnitude smaller in the opaque simulator, and it would be more difficult to accumulate the same amount of advantage during recombination events. This serves as an important indication that computational plant breeding is likely to overestimate breeding results.

Genetic gains of the children population C are shown in **Figure 6** to more clearly analyze the effect of different settings for hybrid breeding. First, we can observe the impacts of the three predictors: the perfect predictor brought the upper limit of the genetic gains, followed by the Bayesian predictor and finally by the phenotypic predictor. This ranking accentuated the need to use genetic prediction to aid breeding. Moreover, when we compare the first column with the third column, and the second column with the fourth column, we find that SCA boosted more genetic gains for the Bayesian predictor, and GCA had greater benefits for the phenotypic predictor. Furthermore, the responses of the transparent and the opaque simulator to the two genomic selections also differed in the first and second row, with the advantages of SCA being only limited to the phenotypic predictor given the transparent simulator. Nevertheless, SCA improved the genetic gains of the Bayesian predictor, making the performance closer to the perfect predictor when using the opaque simulator. These differences underscored the observable benefits of more subtle design of genomic selection and mating on the opaque simulator.

### 3.3 Influences of environmental effect through heritability

**Figure 7:**
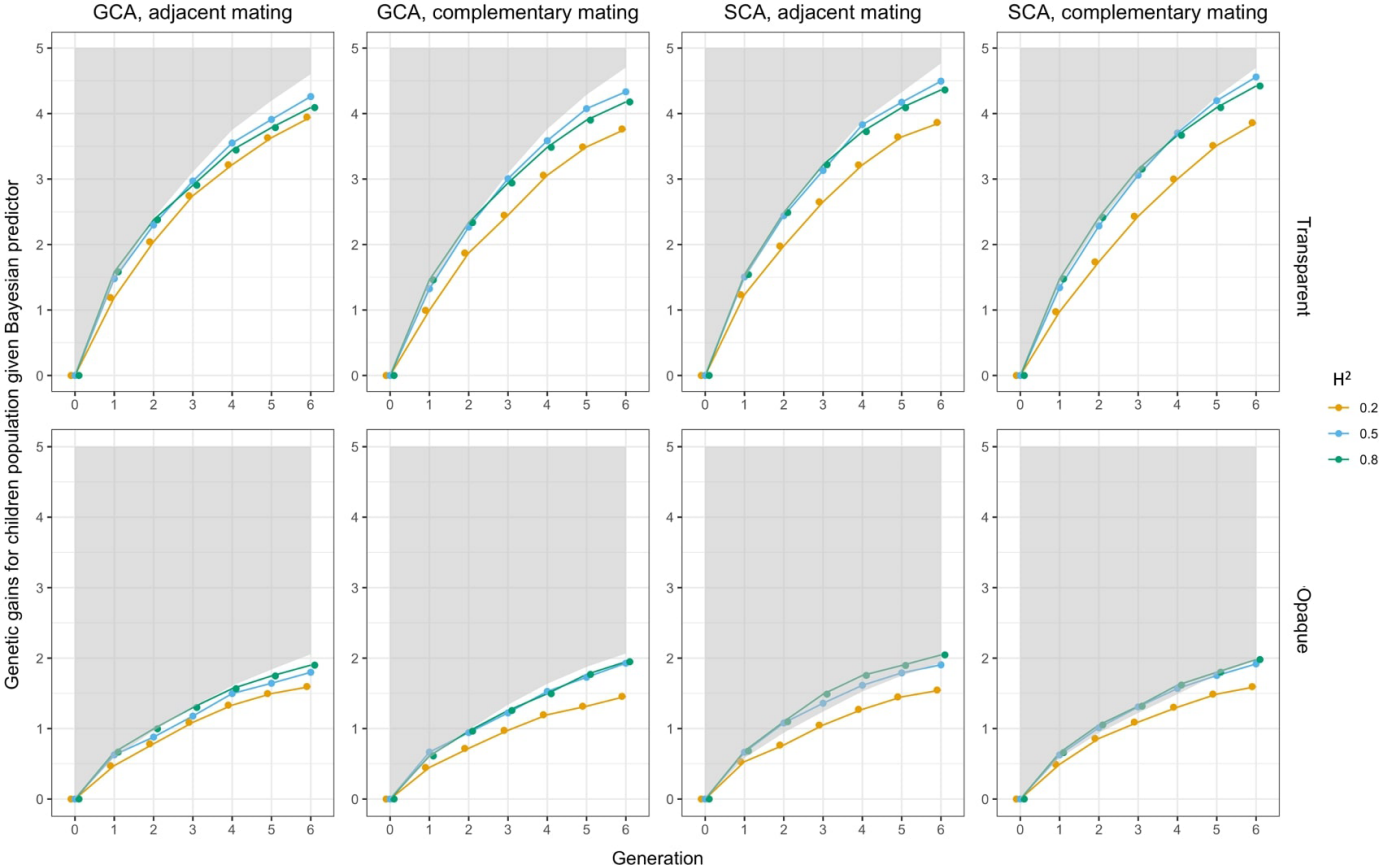
Sensitivity analysis on heritability $H^{2}$ when Bayesian predictor was applied. In each subfigure, the lower bound of the gray shaded area represented the genetic gains of C when using perfect predictor, and the solid lines represented the genetic gains when using Bayesian predictor. Colors indicated the magnitudes of $H^{2}$ .

**Figure 8:**
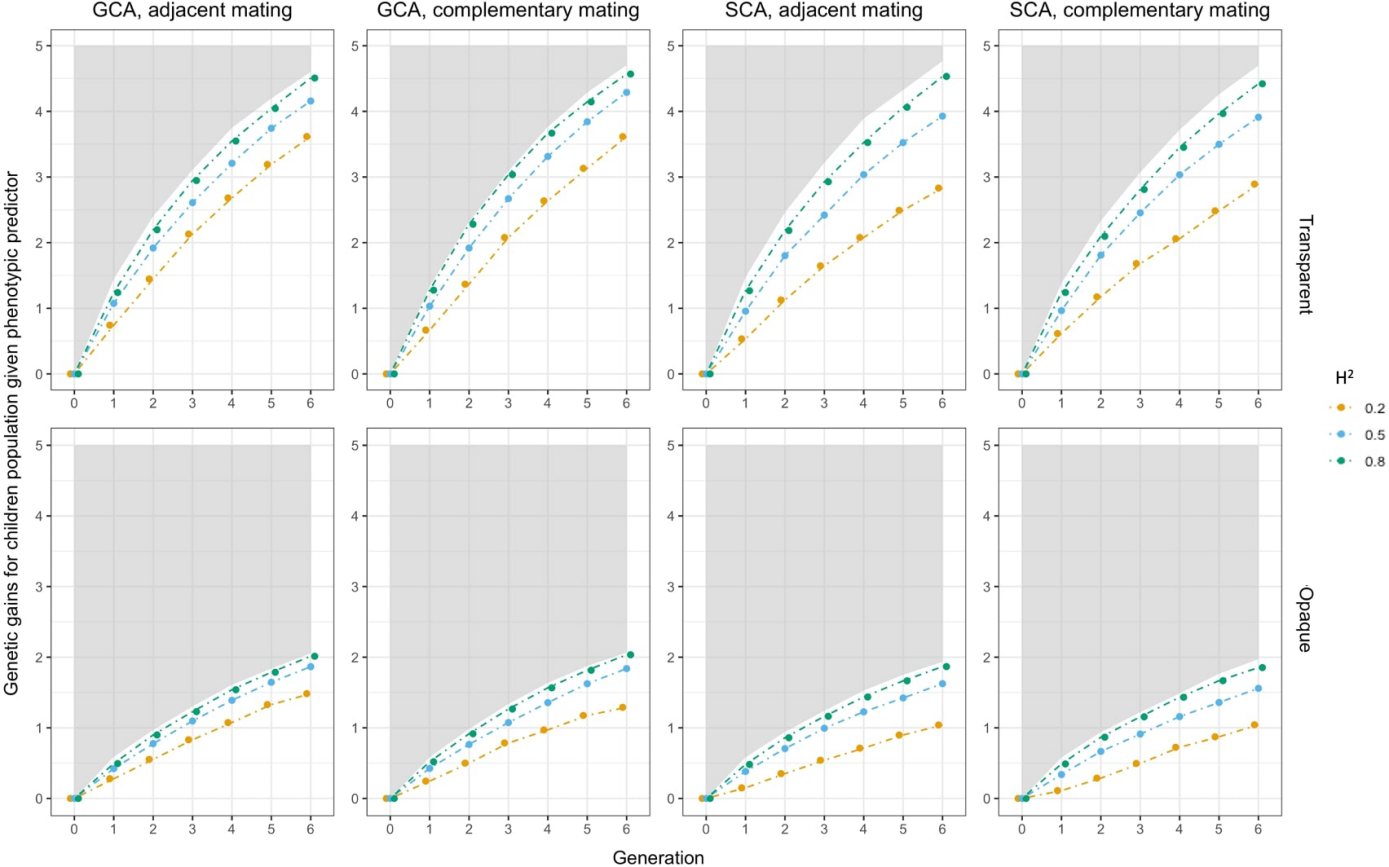
Sensitivity analysis on heritability $H^{2}$ when phenotypic predictor was applied. In each subfigure, the lower bound of the gray shaded area represented the genetic gains of C when using perfect predictor, and the solid lines represented the genetic gains when using phenotypic predictor. Colors indicated the magnitudes of $H^{2}$ .

In real-life production, the influence of the environment on plants cannot be ignored. We use broad-sense heritability $H^{2}$ to adjust the effect of environment on plant phenotypes in this article. Fluctuations in plant phenotypes would affect the prediction accuracy of the Bayesian predictor and the evaluation of the phenotypic predictor, while they have no effect on the perfect predictor, so that we did sensitivity analysis for $H^{2}$ on experiments 5 to 12 and 17 to 24 . Three values of $H^{2}\;\left(0.2,\;0.5,\;\text{and}\;0.8\right)$ were chosen, and the average genetic gains of the children’s population C were recorded as the results. As shown in **Figure 7** for the Bayesian predictor and **Figure 8** for the phenotypic predictor, each subplot showed the performance of the hybrid breeding under the same predictor and different $H^{2}$ . The performance by the perfect predictor was also given as the lower bound of the shaded area in each subplot and was the same as the result from **Figure 6**.

The results showed that genetic gains of both the Bayesian predictor and the phenotypic predictor were amplified with increasing $H^{2}$ . The boundaries between trajectories were clear, and all of them fell below the performance of the perfect predictor in **Figure 8**. For the Bayesian predictor, we noted that the genetic gains given by the transparent simulator were initially led by the case at $H^{2}=0.8$ , and after a few cycles, they were overtaken by the performance at $H^{2}=0.5$ . The opaque simulator, on the other hand, maintained the trend of greater genetic gains for larger $H^{2}$ . Moreover, at $H^{2}=0.5$ and 0.8, the genomic selection SCA improved the genetic gains of the Bayesian predictor even more than the perfect predictor, especially for the opaque simulator. Both signals pointed to the fact that improved prediction accuracy needed to be paired with appropriate and effective genomic selection and mating strategies to improve breeding performance even more. This was true even for imperfect predictions of the opaque simulator.

## 4 Conclusions

In this paper, we extended the concepts of transparent and opaque simulators to RRS-based hybrid breeding and compared the performances of various strategies for genomic prediction, genomic selection, and mating. In previous genomic prediction and selection models, researchers mostly assumed transparent simulators, in which the same set of markers were deployed in both phenotype simulation and genomic prediction. Recently, the concept of opaque simulators was defined in Amini et al. (2021), and similar concepts were independently implemented in the breeding simulation package AlphaSimR (Gaynor et al., 2021). Owing to the opacity and complexity of nature, we believe that opaque simulators are more realistic and appropriate, where the decision-making modules have access to a smaller genotype data than what is used by the simulation modules to produce the phenotype. As such, the use of opaque simulators is expected to help researchers design genomic prediction and genomic selection algorithms that better represent reality and have more robust performance in real-world breeding programs.

The framework also incorporates broad-sense heritability as an adjustment for environmental effects to bring it closer to reality. A sensitivity analysis was performed on the environmental effect $H^{2}$ for both the phenotypic predictor and the Bayesian predictor, which are the two predictors that would be impacted by the varying phenotypic values. One important finding was that even with imperfect genetic prediction results, genomic selection and mating strategies would still potentially benefit hybrid breeding. This may direct us to pay some attention to more sophisticated selection and mating algorithms in future research.

This study is not without its limitations. For example, the proposed framework only considered dominance effects in the simulation modules, but did not explicitly incorporate epistatic effects or genotype-by-environment (G×E) interactions, which also play critical roles in the breeding process. The purpose of this omission was to avoid complex interactions between epistasis or G×E and opaque simulators. After observing the differences between the transparent and opaque simulators, a natural follow-up direction is to introduce epistasis and G×E to make the breeding simulator more realistic.

## Data availability statement

The original contributions presented in the study are included in the article/supplementary material. Further inquiries can be directed to the corresponding author.

## Author contributions

LW and ZZ conceived the project and wrote the manuscript. ZZ coded the algorithms and performed the computational experiments. The authors are grateful to the editor and reviewers, whose feedback greatly improved the quality of this manuscript. All authors contributed to the article and approved the submitted version.
